# Response to: Five methodological considerations for validating LLMs in risk of bias assessment

**DOI:** 10.1017/rsm.2026.10102

**Published:** 2026-06-08

**Authors:** Angelika Eisele-Metzger, Judith-Lisa Lieberum, Markus Toews, Waldemar Siemens, Felix Heilmeyer, Christian Haverkamp, Daniel Boehringer, Joerg J. Meerpohl

**Affiliations:** 1 Institute for Evidence in Medicine, Medical Center – University of Freiburg, Faculty of Medicine, University of Freiburg, Freiburg, Germany; 2 Cochrane Germany, Cochrane Germany Foundation, Freiburg, Germany; 3 Eye Center, Medical Center – University of Freiburg, Faculty of Medicine, University of Freiburg, Freiburg, Germany; 4 Institute for Digitization in Medicine, Medical Center – University of Freiburg, Faculty of Medicine, University of Freiburg, Freiburg, Germany

Dear Editors,

We thank Vihaan Sahu^1^ for the thoughtful letter concerning our article “Exploring the potential of Claude 2 for risk of bias assessment: Using a large language model to assess randomized controlled trials with RoB 2.”^2^ Below, we respond to the methodological considerations raised in the letter.

## 1 Imperfect reference standard—human RoB 2 assessments

We acknowledge that using published Cochrane RoB 2 judgments as a reference standard has its limitations, given the modest inter-rater reliability reported for the tool.^3^ ^,^ ^4^ However, this limitation was already discussed in our article. As we suggested, future work could employ a purpose-built expert reference standard (e.g., from a panel of experts), although this would also require considerably more time and resources. Given the high methodological quality of most Cochrane reviews, the assessments by various Cochrane authors were arguably best suited for our project to establishing an external and feasible reference standard.

## 2 Augmentation rather than replacement

We agree that a “human-in-the-loop” or semiautomated approach may currently be the most practical way to harness the judgments made by a large language model (LLM). Our study did not test such a workflow, but we welcome further investigations. Plausible scenarios include using an LLM for an initial, rough pre-assessment or for flagging potentially critical sections of a report, which are then verified by human reviewers. Likewise, an LLM could assist with extracting or highlighting data relevant to the risk of bias assessment, as suggested in our discussion.

## 3 Information parity between model and human reviewers

In our study, the LLM received the full-text article together with a compressed protocol or registry entry (when available). Human Cochrane reviewers, in contrast, could consult any supplementary material, contact study authors, and examine complete protocols. This consequently resulted in a slight imbalance in the information available to LLMs and human reviewers. However, providing every piece of ancillary information for the 100 randomized controlled trials we examined would have been unfeasible. Moreover, as described in our article, Claude struggled with very long protocols. Therefore, we implemented a refined approach by supplying a compressed version to ensure that the model assessed the study itself rather than an extensive protocol.

## 4 Exploration of performance heterogeneity

Claude’s agreement with human judgments varied across RoB 2 domains, that is, highest Cohen’s κ for Domain 3 (“missing data”), followed by Domain 4 (“outcome measurement”), and lowest for Domain 5 (“selective reporting”) in our main analysis. This suggests that there is likely some heterogeneity in performance across the various RoB-2 domains. As described in our article, we conducted several additional analyses to investigate possible influencing factors, including, e.g., sensitivity analyses using two alternative prompts and one alternative model (Claude 3), subgroup analyses by intervention type (pharmacological vs. other—nonpharmacological, nonsurgical—interventions), and subgroup analyses by the availability of a published protocol or registry entry. Of course, these were not exhaustive. Although κ values fluctuated modestly in our additional analyses, none indicated consistently strong performance. We would therefore urge caution and advise against placing overly strong emphasis on individual κ values.

## 5 Temporal validity and rapid model evolution

We share the concern that evaluations of single LLMs can become outdated quickly in this rapidly evolving field, underscoring the importance of approaches that support reproducibility and continuous reassessments. Our study, conducted in February 2024, contributes an early evaluation within this dynamic landscape. Building on this, we emphasize that future validation studies should be strengthened by incorporating plans for reevaluation and by transparently reporting key details such as model version, run date, prompts, and data used to facilitate replication studies. The RAISE (Responsible Use of AI in Evidence SynthEsis) Statement^5^ ^,^ ^6^ provides valuable guidance in this regard. Complementary initiatives, including the Digital Evidence Synthesis Tool Evaluations^7^ and our currently ongoing living scoping review,^8^ are actively advancing the coordination and tracking of evaluation efforts.

We appreciate Sahu’s insightful appraisal and his constructive proposals. We look forward to further constructive discourse and collaboration with the systematic review community.

## Data Availability

Data sharing is not applicable to this article.
